# Hypothesis: AA amyloidosis is a factor causing systemic complications after coronavirus disease

**DOI:** 10.1080/19336896.2021.1910468

**Published:** 2021-04-20

**Authors:** Alexey P. Galkin

**Affiliations:** a St. Petersburg Branch, Vavilov Institute of General Genetics, St. Petersburg, Russian Federation; b Department of Genetics and Biotechnology, St. Petersburg State University, St. Petersburg, Russian Federation

**Keywords:** Coronavirus, COVID-19, serum amyloid A, AA amyloidosis, chronic inflammation

## Abstract

The severe course of COVID-19 causes systemic chronic inflammation and thrombosis in a wide variety of organs and tissues. The nature of these inflammations remains a mystery, although they are known to occur against the background of a high level of cytokine production. The high level of cytokines provokes overproduction of the Serum amyloid A (SAA) protein. Moreover, the number of studies has shown that the severe COVID-19 causes SAA overproduction. The authors of these works regard a high level of SAA exclusively as a biomarker of COVID-19. However, it should be borne in mind that overproduction of SAA can cause systemic AA amyloidosis. SAA forms cytotoxic amyloid deposits in various organs and induces inflammation and thrombosis. The consequences of COVID-19 infection are surprisingly similar to the clinical picture that is observed in AA amyloidosis. Here I present the hypothesis that AA amyloidosis is a factor causing systemic complications after coronavirus disease.

Identification of factors that cause systemic complications after coronavirus disease (COVID-19) appears to be the next global challenge after vaccine development. Over two million people have already died from COVID-19, and the end of the pandemic is very difficult to predict. Severe course of COVID-19 causes systemic complications of unclear aetiology. The patients who have suffered severe forms of this viral disease develop inflammation of the lungs, kidneys, heart, gastrointestinal stroke, peripheral and central nervous systems [1]. The nature of these inflammations remains a mystery, although they are known to occur against the background of a high level of cytokine production [2]. In addition, such patients frequently suffer from thrombosis that leads to cardiovascular and brain pathologies [3,4].

The consequences of COVID-19 infection are surprisingly similar to the clinical picture that is observed in systemic AA amyloidosis (AA stands for amyloid A). This type of amyloidosis is associated with abnormal aggregation of the Serum amyloid A (SAA) protein [5]. SAA is involved in immune regulation and functions as an opsonin for bacterial phagocytosis. This protein also regulates reverse cholesterol transport from injured tissues [5]. Chronic infections (such as tuberculosis, osteomyelitis and bronchiectasis), autoimmune diseases and chronic inflammatory disorders increase the level of production of SAA manyfold [6]. Various types of tumour and chronic viral infections, such as hepatitis B and HIV-1, are also capable of causing AA amyloidosis [7–9]. Overproduction of this protein is induced by an increased level of cytokines [10]. Under these conditions, the N-terminal fragment is cleaved from SAA and forms cytotoxic amyloid deposits in the extracellular space of various tissues and organs [5]. These events induce inflammation and blood vessel thrombosis [11]. The cytotoxic effect of amyloid deposits increases the level of cytokines, which causes overproduction of SAA [10]. Thus, a closed cycle of mutual stimulation of cytokines and SAA is formed. Importantly, a number of medical reports convincingly show that coronavirus causes SAA protein overproduction [12–15] and that extremely high serum SAA level correlates with poor disease prognosis [16]. All these authors regard a high level of SAA exclusively as a biomarker of COVID-19 infection.

Generalization of all these published data suggests a hypothesis according to which AA amyloidosis is a factor causing systemic pathologies after a severe form of coronavirus disease.

All these data are aligned in the following logical chain:

1. The severe course of coronavirus disease causes an increased production of cytokines, and sometimes a cytokine storm [16];
2. High cytokine level provokes SAA overproduction [10];
3. SAA overproduction causes formation of amyloid aggregates that are deposited on the walls of blood vessels in various organs and tissues [5];
4. Cytotoxic protein aggregates cause inflammation of the affected tissues [5] and vascular thrombosis [11].

Only one element in this hypothesis remains untested. It is necessary to prove experimentally that SAA overproduction against the background of a severe course of COVID-19 disease does lead to formation of amyloid deposits. This scenario appears to be most likely. As mentioned above, chronic inflammations after bacterial and viral infections are known to cause AA amyloidosis [6]. Confirmation of AA amyloidosis can be obtained using standard biopsy followed by immunodetection and Congo red staining of tissue samples. The binding specificity of Congo red is identified by the presence of yellow-green birefringence in polarized light [17].

If this hypothesis is verified, therapy aimed at suppressing inflammation and reducing the level of SAA production will be required. It is important to note that AA amyloidosis is treatable if diagnosed in time. Various therapies have been used successfully for treatment of this type of amyloidosis [5,18–20]. It should be borne in mind that the choice of therapy depends on the disease that caused amyloidosis, patient’s history, degree of organ damage and serum SAA concentration.

The hypothesis postulating that AA amyloidosis is a factor causing systemic pathologies after coronavirus disease appears to be very convincing, although the pathogenesis may also be associated with other factors. In particular, these complications could presumably be related to other types of systemic amyloidosis. In any case, the risk of AA amyloidosis induction in severe COVID-19 cases is quite high. Verification of this hypothesis appears to be an urgent task as identification of the factors causing systemic complications after COVID-19 could help to save many lives.

## References

[1] Machhi J, Herskovitz J, Senan AM, et al. The natural history, pathobiology, and clinical manifestations of SARS-CoV-2 infections. J Neuroimmune Pharmacol. 2020 9;15(3):359–386. 32696264 10.1007/s11481-020-09944-5 PMC7373339

[2] Dhakal BP, Sweitzer NK, Indik JH, et al. SARS-CoV-2 infection and cardiovascular disease: COVID-19 heart. Heart Lung Circ. 2020 7;29(7):973–987. 32601020 10.1016/j.hlc.2020.05.101 PMC7274628

[3] Klok FA, Kruip MJHA, van der Meer NJM, et al. Incidence of thrombotic complications in critically ill ICU patients with COVID-19. Thromb Res. 2020;191:145–147. 32291094 10.1016/j.thromres.2020.04.013 PMC7146714

[4] Wang J, Hajizadeh N, Moore EE, et al. Tissue plasminogen activator (tPA) treatment for COVID-19 associated acute respiratory distress syndrome (ARDS): a case series. J Thromb Haemost. 2020 7;18(7):1752–1755. 32267998 10.1111/jth.14828 PMC7262152

[5] Westermark GT, Fändrich M, Westermark P. AA amyloidosis: pathogenesis and targeted therapy. Annu Rev Pathol. 2015;10:321–344. 25387054 10.1146/annurev-pathol-020712-163913

[6] Deshayes S, Aouba A, Grateau G, et al. Infections and AA amyloidosis: an overview. Int J Clin Pract. 2020 12;23:e13966. 10.1111/ijcp.13966 33368925

[7] Saha A, Theis JD, Vrana JA, et al. AA amyloidosis associated with hepatitis B. Nephrol Dial Transplant. 2011 7;26(7):2407–2412. 21543651 10.1093/ndt/gfr224

[8] Sack GH Jr. Serum amyloid A - a review. Mol Med. 2018 8 30;24(1):46. 30165816 10.1186/s10020-018-0047-0 PMC6117975

[9] Borg J, Buttigieg J, Holwill S, et al. Treatment of renal AA-Amyloidosis associated with human immunodeficiency virus infection: a case report. CEN Case Rep. 2021 2;10(1):88–93. 32889702 10.1007/s13730-020-00525-2 PMC7829295

[10] Blank N, Lorenz HM. Diagnostik und Therapie der AA-Amyloidose [Diagnostics and therapy of AA amyloidosis]. Pathologe. 2009 5;30(3):219–225. 19333604 10.1007/s00292-009-1140-5

[11] Srkalovic G, Cameron MG, Deitcher SR, et al. Incidence and risk factors of venous thromboembolism (VTD) in patients with amyloidosis. Int Semin Surg Oncol. 2005;2:17. 16138931 10.1186/1477-7800-2-17 PMC1232862

[12] Liu SL, Wang SY, Sun YF, et al. Expressions of SAA, CRP, and FERR in different severities of COVID-19. Eur Rev Med Pharmacol Sci. 2020 11;24(21):11386–11394. 33215460 10.26355/eurrev_202011_23631

[13] Tjendra Y, Al Mana AF, Espejo AP, et al. Predicting disease severity and outcome in COVID-19 patients: a review of multiple biomarkers. Arch Pathol Lab Med. 2020 12 1;144(12):1465–1474. 32818235 10.5858/arpa.2020-0471-SA

[14] Chen M, Wu Y, Jia W, et al. The predictive value of serum amyloid A and C-reactive protein levels for the severity of coronavirus disease 2019. Am J Transl Res. 2020 8 15;12(8):4569–4575. 32913530 PMC7476119

[15] Haroun RA, Osman WH, Eessa AM. Interferon-γ-induced protein 10 (IP-10) and serum amyloid A (SAA) are excellent biomarkers for the prediction of COVID-19 progression and severity. Life Sci. 2021 3 15;269:119019. 33454365 10.1016/j.lfs.2021.119019 PMC7832132

[16] Li H, Xiang X, Ren H, et al. Serum Amyloid A is a biomarker of severe Coronavirus disease and poor prognosis. J Infect. 2020 6;80(6):646–655. 32277967 10.1016/j.jinf.2020.03.035 PMC7141628

[17] Merlini G, Saraiva MJM, Sekijima Y, et al. Amyloid nomenclature 2018: recommendations by the international society of amyloidosis (ISA) nomenclature committee. Amyloid. 2018 12;25(4):215–219. 30614283 10.1080/13506129.2018.1549825

[18] Lachmann HJ, Goodman HJ, Gilbertson JA, et al. Natural history and outcome in systemic AA amyloidosis. N Engl J Med. 2007 6 7;356(23):2361–2371. 17554117 10.1056/NEJMoa070265

[19] Nuvolone M, Merlini G. Systemic amyloidosis: novel therapies and role of biomarkers. Nephrol Dial Transplant. 2017 5 1;32(5):770–780. 27540044 10.1093/ndt/gfw305

[20] Papa R, Lachmann HJ. Secondary, AA, Amyloidosis. Rheum Dis Clin North Am. 2018 11;44(4):585–603. 30274625 10.1016/j.rdc.2018.06.004

